# The Evolution of Neoadjuvant Treatment for Resectable Esophageal Squamous Cell Carcinoma: Past Achievements, Current Standards, and Future Perspectives

**DOI:** 10.3390/cancers18203350

**Published:** 2026-10-16

**Authors:** Naoto Ujiie, Yusuke Taniyama, Chiaki Sato, Hiroshi Okamoto, Yohei Ozawa, Hirotaka Ishida, Michiaki Unno, Takashi Kamei

**Affiliations:** Department of Surgery, Tohoku University, 1-1 Seiryo-machi, Aoba-ku, Sendai 980-8574, Miyagi, Japan; yusuke.taniyama.b8@tohoku.ac.jp (Y.T.); schiaki@tohoku.ac.jp (C.S.); hiroshi.okamoto.b2@tohoku.ac.jp (H.O.); yohei.ozawa.c1@tohoku.ac.jp (Y.O.); hirotaka.ishida.e1@tohoku.ac.jp (H.I.); michiaki.unno.e5@tohoku.ac.jp (M.U.); takashi.kamei.a8@tohoku.ac.jp (T.K.)

**Keywords:** esophageal squamous cell carcinoma, neoadjuvant chemotherapy, neoadjuvant chemoradiotherapy, immunotherapy, response-adapted treatment

## Abstract

Neoadjuvant treatment has become an integral component of multidisciplinary management for resectable esophageal squamous cell carcinoma (ESCC). Over recent decades, treatment has evolved from surgery alone to postoperative chemotherapy, neoadjuvant chemotherapy, intensified systemic therapy, neoadjuvant chemoradiotherapy, and, more recently, the incorporation of immune checkpoint inhibitors into curative-intent treatment. This evolution has been driven by persistent challenges including systemic recurrence, locoregional failure, treatment-related toxicity, and heterogeneous treatment response. This review traces the evolution of neoadjuvant treatment for resectable ESCC through landmark clinical trials that have shaped current practice. We discuss the transition from postoperative to neoadjuvant chemotherapy, the development of intensified chemotherapy and neoadjuvant chemoradiotherapy, and the emergence of immunotherapy, distinguishing the established role of adjuvant nivolumab from the emerging role of neoadjuvant immunochemotherapy. We then examine the unresolved relative roles of these strategies and the challenges in treatment selection. Finally, we discuss future directions centered on accurate response assessment, circulating tumor DNA-based molecular residual disease assessment, and response-adapted treatment, with particular emphasis on organ-preserving strategies after a clinical complete response. Although active surveillance has emerged as a potential alternative to immediate surgery in carefully selected patients, accurate identification of residual disease and appropriate surveillance remain essential. Future advances may therefore shift the focus from treatment intensification alone toward response-adapted strategies that can maintain oncological safety while potentially enabling organ preservation.

## Simple Summary

Esophageal squamous cell carcinoma is often diagnosed at a stage when surgery alone is insufficient to prevent recurrence. Over the past decades, treatment before surgery has evolved from chemotherapy to more intensive chemotherapy, chemoradiotherapy, and, more recently, immunotherapy. These advances have expanded treatment options, but important questions remain regarding the optimal neoadjuvant strategy and how treatment response should influence subsequent management. In this review, we summarize major clinical trials that have shaped neoadjuvant treatment and discuss the strengths and limitations of current approaches. We also examine emerging strategies for assessing residual disease, including circulating tumor DNA and molecular residual disease assessment, with particular attention to response-adapted management and the potential for organ preservation in patients achieving a clinical complete response. These developments may help enable more precise selection of patients for continued surveillance or surgery.

## 1. Introduction

Esophageal squamous cell carcinoma (ESCC) remains a major cause of cancer-related morbidity and mortality worldwide, with a particularly high disease burden in East Asia. Although surgical resection offers the possibility of cure for patients with resectable disease, postoperative recurrence remains common because of both locoregional failure and occult systemic disease. These limitations provided the rationale for integrating systemic and locoregional therapies into multimodal treatment strategies [1,2].

Over the past three decades, perioperative treatment has evolved from postoperative chemotherapy to preoperative chemotherapy, neoadjuvant chemoradiotherapy (nCRT), intensified systemic therapy, and, more recently, immune checkpoint inhibitor-based treatment [3,4,5,6,7,8,9,10,11]. Postoperative chemotherapy was followed by evidence supporting preoperative chemotherapy in Japan [3,4], while nCRT emerged as an alternative approach to improve locoregional control [5,6]. The JCOG1109 trial subsequently demonstrated a survival benefit with intensified triplet chemotherapy over conventional doublet chemotherapy [7]. Meanwhile, immune checkpoint inhibition, initially established in advanced ESCC [8], was introduced into curative-intent treatment with adjuvant nivolumab after nCRT and surgery [9], followed by increasing evidence for neoadjuvant immunochemotherapy [10,11].

This evolution has not followed a simple linear progression toward a single optimal treatment. Rather, different strategies have emerged to address distinct unresolved challenges, including systemic recurrence, locoregional control, treatment intensity, and heterogeneity in treatment response. The evidence base has also varied across clinical settings because of differences in disease epidemiology, histological distribution, treatment practices, and trial populations. Accordingly, the roles of neoadjuvant chemotherapy (nCT), nCRT, and emerging immunotherapy-based approaches must be considered within their respective clinical contexts.

In this review, we trace the evolution of neoadjuvant treatment for resectable ESCC from surgery alone to contemporary multimodal and emerging precision approaches. We examine the landmark trials that have shaped current treatment, the unresolved relative roles of nCT and nCRT, and the emerging role of immunotherapy in the curative-intent setting. We then discuss future strategies, including biomarker-guided treatment selection, circulating tumor DNA (ctDNA)-based molecular residual disease (MRD) assessment, early response assessment, and response-adapted treatment.

Although the primary focus of this review is neoadjuvant treatment, selected postoperative and response-adapted strategies are discussed when they are directly relevant to its evolution. These include adjuvant nivolumab after nCRT and surgery, MRD assessment, and active surveillance after a clinical complete response. These approaches are not considered neoadjuvant treatments themselves but illustrate how management after neoadjuvant therapy may increasingly contribute to response-adapted treatment strategies.

Relevant literature was identified through searches of the PubMed database using terms related to esophageal squamous cell carcinoma, neoadjuvant chemotherapy, chemoradiotherapy, and immunotherapy. No restrictions were imposed on the publication period. Particular emphasis was placed on landmark randomized controlled trials that established or challenged neoadjuvant treatment strategies, as well as recent prospective studies investigating immunotherapy, ctDNA/MRD assessment, and organ-preserving approaches. Relevant publications were selected based on their clinical relevance to the evolution of neoadjuvant treatment for resectable ESCC. This narrative review was not designed as a systematic review or meta-analysis.

## 2. Why Surgery Alone Was Not Enough

Esophagectomy with regional lymphadenectomy has long been the principal curative treatment for resectable ESCC. However, postoperative recurrence remains common, particularly in patients with locally advanced disease. In Japanese series, recurrence after radical surgery has been reported in approximately 28–47% of patients, with both lymph-node or locoregional recurrence and distant metastasis contributing to treatment failure [12,13]. These patterns indicate that clinically resectable ESCC may already harbor occult systemic disease at the time of surgery [13,14].

The limitation of surgery alone is therefore not simply inadequate local control. Although esophagectomy can remove the primary tumor and clinically detectable regional disease, it cannot eradicate microscopic systemic disease that remains undetectable by conventional staging. This provided the rationale for incorporating systemic therapy into curative-intent treatment [12,15,16,17].

The initial strategy was postoperative chemotherapy. In the JCOG9204 randomized trial, postoperative cisplatin plus fluorouracil (CF) improved 5-year disease-free survival (DFS) compared with surgery alone (55% vs. 45%), although the difference in overall survival (OS) was not statistically significant [3]. The benefit was particularly evident in patients with pathological lymph-node metastases [3]. However, postoperative chemotherapy has practical limitations: the physiological stress of esophagectomy, postoperative complications, impaired oral intake, and nutritional deterioration may compromise treatment delivery [18]. Moreover, systemic therapy is delayed until after surgery, leaving occult disease untreated during the preoperative period [15,19].

These limitations prompted a shift toward treatment before surgery. Preoperative chemotherapy allows systemic treatment to be delivered before the physiological burden of esophagectomy, treats occult systemic disease earlier, and provides an opportunity to assess treatment response before surgery [14,16,19,20]. The JCOG9907 trial directly compared preoperative and postoperative CF chemotherapy in patients with clinical stage II/III thoracic ESCC and demonstrated superior OS with preoperative treatment (5-year OS, 55% vs. 43%; hazard ratio (HR), 0.73) [4]. These findings established preoperative chemotherapy as the standard treatment for stage II/III ESCC in Japan [4,21].

**Figure 1:**
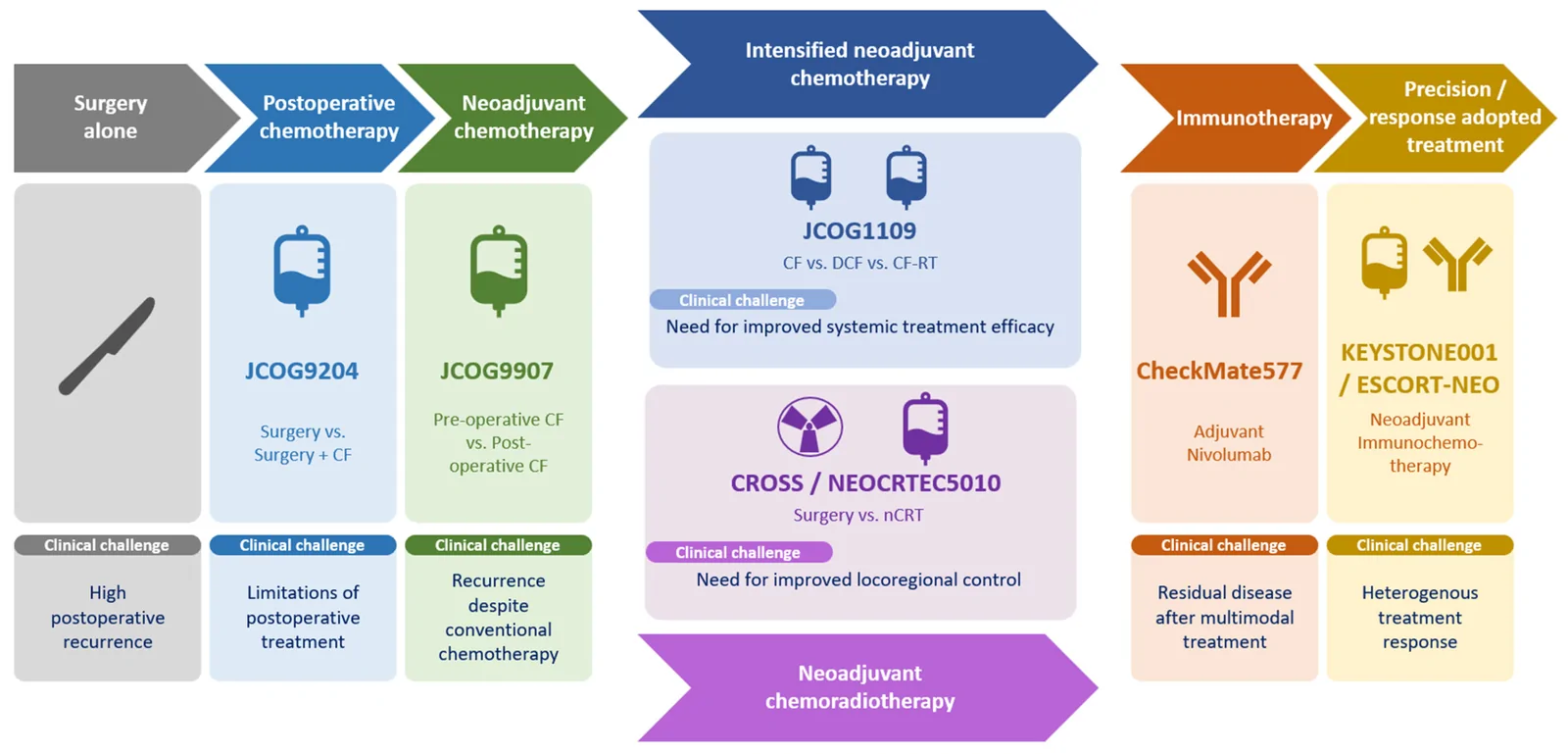
Evolution of neoadjuvant treatment for resectable esophageal squamous cell carcinoma. The evolution of perioperative treatment for resectable esophageal squamous cell carcinoma has been driven by unresolved limitations of preceding treatment strategies. Treatment progressed from surgery alone to postoperative chemotherapy, neoadjuvant chemotherapy, intensified neoadjuvant treatment, and neoadjuvant chemoradiotherapy, followed by the incorporation of immune checkpoint inhibitors into curative-intent treatment. Current development is shifting toward biomarker-driven and response-adapted strategies that integrate molecular residual disease assessment. Thus, the evolution of treatment has been non-linear, with parallel strategies addressing systemic disease control, locoregional control, and treatment-related limitations. CF, cisplatin plus fluorouracil; DCF, docetaxel, cisplatin, and fluorouracil; ESCC, esophageal squamous cell carcinoma; nCRT, neoadjuvant chemoradiotherapy; PD-1, programmed cell death protein 1.

Preoperative chemotherapy, however, did not eliminate recurrence or resolve the optimal balance between systemic and locoregional treatment. These unresolved issues prompted further treatment intensification, including more intensive chemotherapy and nCRT, while the subsequent introduction of immune checkpoint inhibitors (ICIs) provided a new approach to treatment intensification. The major therapeutic transitions in this evolution are illustrated in Figure 1.

## 3. The Establishment of Neoadjuvant Chemotherapy

The transition from postoperative to preoperative chemotherapy represented a major shift in the systemic treatment of resectable ESCC. JCOG9204 established the benefit of postoperative chemotherapy in reducing recurrence after surgery, while JCOG9907 subsequently demonstrated superior survival with the same CF regimen when administered before surgery [3,4]. Preoperative chemotherapy therefore became a central component of curative-intent treatment for locally advanced ESCC in Japan. The next question was whether its efficacy could be further improved by intensifying systemic treatment, leading to the JCOG1109 NExT trial [7].

### 3.1. From Postoperative Chemotherapy to Neoadjuvant Chemotherapy

The JCOG9204 trial provided the initial randomized evidence supporting postoperative systemic chemotherapy [3]. In this phase III trial, 242 patients with localized squamous cell carcinoma of the thoracic esophagus were randomly assigned to surgery alone or surgery followed by two courses of CF. Postoperative chemotherapy significantly improved 5-year DFS compared with surgery alone (55% vs. 45%; *p* = 0.037), although the difference in 5-year OS was not statistically significant (61% vs. 52%; *p* = 0.13) [3]. The reduction in recurrence was particularly evident in patients with pathological lymph-node metastases, supporting the role of systemic chemotherapy in reducing postoperative relapse [3].

The JCOG9907 trial subsequently addressed whether chemotherapy should be administered before rather than after surgery [4]. Patients with clinical stage II/III thoracic ESCC were randomly assigned to two courses of CF administered preoperatively or postoperatively. Preoperative chemotherapy significantly improved OS, with a 5-year survival rate of 55% compared with 43% after postoperative chemotherapy (HR, 0.73; *p* = 0.04) [4]. Because the chemotherapy regimen was identical in both groups, the trial provided direct evidence that treatment timing itself could influence outcomes. These findings established preoperative CF as the standard treatment for stage II/III ESCC in Japan [4,21,22].

The advantages of preoperative treatment extend beyond timing. Chemotherapy can be delivered before the physiological stress of esophagectomy, occult micrometastatic disease can be treated earlier, and tumor response can provide an indication of treatment sensitivity before surgery [19,20,23]. However, despite the establishment of preoperative CF, recurrence remained common after nCT and surgery. This raised the next question in treatment evolution: whether increasing systemic treatment intensity could further improve outcomes.

### 3.2. Intensifying Neoadjuvant Chemotherapy: The JCOG1109 NExT Trial

The JCOG1109 NExT trial evaluated whether neoadjuvant treatment could be intensified beyond conventional CF chemotherapy [7]. This randomized phase III trial enrolled 601 patients with locally advanced ESCC and compared three preoperative strategies: CF chemotherapy, triplet chemotherapy with docetaxel, cisplatin, and fluorouracil (DCF), and CF combined with radiotherapy (CF-RT) [7]. The three-arm design allowed systemic and locoregional intensification to be evaluated within the same trial.

DCF significantly improved OS compared with CF, with 3-year OS rates of 72.1% and 62.6%, respectively (HR, 0.68; *p* = 0.006) [7]. In contrast, CF-RT did not significantly improve OS compared with CF (68.3%; HR, 0.84; *p* = 0.12) [7]. Thus, within the JCOG1109 study population, increasing systemic chemotherapy intensity improved survival, whereas the addition of radiotherapy to CF did not demonstrate a significant survival advantage.

The benefit of DCF was accompanied by increased treatment-related toxicity. Grade ≥ 3 febrile neutropenia occurred in 16% of patients receiving DCF compared with 1% receiving CF [7]. The trial therefore demonstrated both the potential benefit and the cost of systemic treatment intensification, underscoring the need to balance efficacy with treatment tolerability.

The results of JCOG1109 also highlighted an important distinction between systemic and locoregional treatment intensification. The lack of a significant survival benefit with CF-RT did not exclude a role for nCRT, because the trial evaluated a specific CF-based regimen and radiation strategy rather than nCRT as a class [7]. Rather, JCOG1109 provided evidence that more intensive systemic chemotherapy could improve outcomes in locally advanced ESCC while leaving the incremental value of radiotherapy over contemporary nCT unresolved.

The evolution from postoperative CF to preoperative CF and subsequently to DCF thus reflects successive attempts to reduce recurrence, optimize treatment timing, and increase systemic treatment efficacy. However, intensified chemotherapy did not fully address the question of whether greater locoregional tumor control could provide additional benefit. This issue forms the basis for the parallel development of nCRT, discussed in the next section. The major randomized trials shaping the evolution of neoadjuvant treatment for resectable ESCC are summarized in Table 1.

## 4. The Role of Neoadjuvant Chemoradiotherapy

The establishment of nCT addressed the systemic component of recurrence, but locoregional failure remained an important concern in locally advanced ESCC. nCRT therefore emerged as a complementary strategy that combined systemic therapy with radiotherapy to enhance tumor regression and locoregional control before surgery [27,28].

Regional differences in neoadjuvant treatment strategies for esophageal cancer reflect the historical development of clinical evidence and established treatment practices. In Japan, preoperative chemotherapy became the standard approach for resectable locally advanced ESCC following the JCOG9907 trial, which demonstrated superior survival with preoperative compared with postoperative chemotherapy [4]. The subsequent JCOG1109 NExT trial further supported systemic treatment intensification by demonstrating improved OS with neoadjuvant DCF compared with conventional CF chemotherapy [7]. In contrast, nCRT gained a central role in Western practice following the CROSS trial, which demonstrated improved survival and R0 resection rates compared with surgery alone [5]. The NEOCRTEC5010 trial subsequently provided randomized evidence supporting nCRT specifically in locally advanced ESCC [6]. These differences should not be interpreted as evidence that one strategy is universally superior to the other, because the trials differed in their populations, treatment regimens, and comparators. Rather, they reflect distinct evidence-based treatment pathways, underscoring the need to evaluate the relative roles of neoadjuvant chemotherapy and nCRT in the context of the relevant clinical setting.

### 4.1. The Emergence of Neoadjuvant Chemoradiotherapy

The CROSS trial was a landmark study establishing nCRT as an effective multimodal treatment for resectable esophageal cancer [5,29]. In this multicenter randomized phase III trial, patients with potentially curable esophageal or gastroesophageal junction cancer received weekly carboplatin and paclitaxel with 41.4 Gy of radiotherapy over 5 weeks followed by surgery, or surgery alone [5]. nCRT significantly improved OS (median, 49.4 vs. 24.0 months; HR, 0.66; *p* = 0.003) and increased the R0 resection rate from 69% to 92% [5]. Although the trial included both adenocarcinoma and squamous cell carcinoma, the long-term survival benefit was particularly pronounced in the squamous cell carcinoma subgroup (HR, 0.48; *p* = 0.008) [29]. Ten-year follow-up further confirmed the durability of the survival benefit [30].

Because CROSS included a histologically heterogeneous population and used a chemoradiotherapy regimen that differed from those commonly used in East Asia, its applicability to ESCC in other clinical settings required further evaluation. The NEOCRTEC5010 trial subsequently provided histology-specific evidence in locally advanced ESCC [6,31]. In this randomized phase III trial, nCRT with vinorelbine plus cisplatin and 40 Gy of radiotherapy followed by surgery improved OS compared with surgery alone (HR, 0.74; *p* = 0.03), with 5-year OS rates of 59.9% and 49.1%, respectively [31]. Together, CROSS and NEOCRTEC5010 established that adding radiotherapy to preoperative treatment can improve survival compared with surgery alone in selected patients with locally advanced ESCC [5,6,27,28,29,31].

### 4.2. Does More Locoregional Treatment Improve Outcomes?

Once nCRT had demonstrated benefit over surgery alone, the more clinically relevant question became whether radiotherapy provided an additional benefit over nCT alone. Because both strategies already incorporate systemic therapy, this comparison directly addresses the incremental value of radiotherapy beyond chemotherapy [32,33].

The NeoRes trial directly compared nCT with nCRT in 181 patients with resectable esophageal or gastroesophageal junction cancer, including 50 patients with squamous cell carcinoma [24]. nCRT significantly increased complete histological response and R0 resection rates, but this greater pathological tumor regression did not translate into improved OS [24]. Moreover, health-related quality-of-life analyses showed greater treatment-related symptoms with nCRT, including more pronounced odynophagia after neoadjuvant treatment and a greater symptom burden during follow-up [25]. Thus, greater local tumor regression was not necessarily accompanied by a survival benefit and had to be considered in the context of treatment burden.

The CMISG1701 trial subsequently addressed this question specifically in ESCC. In this randomized phase III trial, 264 patients with cT3–4aN0–1M0 disease received nCRT or nCT followed by minimally invasive esophagectomy [26]. nCRT produced a substantially higher pathological complete response (pCR) rate than nCT (27.7% vs. 2.9%; *p* < 0.001), but OS did not differ significantly between the groups (3-year OS, 64.1% vs. 54.9%; HR, 0.82; *p* = 0.28) [26]. Although pathological response after nCRT is prognostic, a higher pathpCR rate does not necessarily establish the survival superiority of one neoadjuvant strategy over another [34].

Pathological response assessment should also extend beyond regression of the primary tumor to include the response of regional lymph nodes. A meta-analysis of 14 retrospective studies involving 3212 patients with esophageal cancer found that complete lymph node regression was associated with improved OS and DFS, whereas nodal downstaging was associated with improved overall and cancer-specific survival [35]. These findings highlight the complementary prognostic information provided by nodal response and support its consideration alongside primary-tumor regression when evaluating pathological outcomes and postoperative risk. However, these associations should be interpreted as prognostic rather than definitive evidence of differential benefit from a particular neoadjuvant treatment strategy.

Taken together, these trials highlight the distinction between improving pathological response and improving long-term survival. Trials comparing nCRT with surgery alone established the benefit of adding multimodal treatment, whereas trials comparing nCRT with nCT evaluate the incremental contribution of radiotherapy after systemic therapy has already been incorporated. The latter is a more stringent comparison, and the available evidence remains heterogeneous [32,33,36]. Differences in tumor stage, histology, chemotherapy regimen, radiation dose and field, surgical approach, and perioperative management further limit direct comparisons across trials conducted in different settings [32,33,36].

### 4.3. The Current Position of Neoadjuvant Chemoradiotherapy

The accumulated evidence supports nCRT as an effective neoadjuvant strategy, particularly for achieving substantial tumor regression and locoregional control. However, its superiority over contemporary nCT has not been consistently demonstrated [5,6,24,25,26,29,31,32,33,34,36]. This distinction is particularly relevant in ESCC, where intensified systemic chemotherapy, such as DCF, provides an alternative approach to treatment intensification.

nCRT therefore represents an important but non-linear branch in the evolution of neoadjuvant treatment for ESCC. Its development addressed the unresolved problem of locoregional control rather than simply increasing systemic treatment intensity. The remaining challenge is to identify patients in whom the incremental benefit of radiotherapy justifies its additional treatment burden. The emergence of ICIs subsequently introduced a different approach to treatment intensification, aimed at enhancing antitumor immunity rather than further increasing cytotoxic treatment intensity.

## 5. The Emergence of Immunotherapy

The development of nCT and nCRT improved the treatment of resectable ESCC, but recurrence remained a major challenge despite curative-intent multimodal therapy. Further intensification of cytotoxic treatment or addition of radiotherapy did not eliminate systemic or locoregional recurrence and could increase treatment burden. These limitations prompted interest in a different strategy: enhancing the antitumor immune response rather than further increasing cytotoxic treatment intensity [37,38].

ICIs, particularly antibodies targeting programmed cell death protein 1 (PD-1) or programmed cell death 1 ligand 1 (PD-L1), had already demonstrated clinical activity in advanced ESCC. In ATTRACTION-3, nivolumab improved OS compared with investigator’s-choice chemotherapy in previously treated advanced ESCC (median, 10.9 vs. 8.4 months; HR, 0.77; *p* = 0.019) [8]. KEYNOTE-181 similarly demonstrated an OS benefit with pembrolizumab over chemotherapy in advanced esophageal cancer, particularly in patients with PD-L1 combined positive score (CPS) ≥ 10 [39]. These findings provided the clinical basis for evaluating immune checkpoint blockade earlier in the disease course [37,38].

### 5.1. CheckMate 577: Establishing Immunotherapy in Curative-Intent Treatment

The phase III CheckMate 577 trial provided an important bridge between immunotherapy in advanced disease and its incorporation into curative-intent treatment [9]. Although it did not evaluate neoadjuvant immunotherapy, it demonstrated that adjuvant nivolumab could reduce recurrence after neoadjuvant chemoradiotherapy and surgery. The trial enrolled patients with resected stage II or III esophageal or gastroesophageal junction cancer who had received nCRT, achieved an R0 resection, and had residual pathological disease. Adjuvant nivolumab significantly prolonged DFS compared with placebo (median, 22.4 vs. 11.0 months; HR, 0.69; *p* < 0.001) [9]. In the prespecified histological subgroup analysis, the DFS benefit was also observed in patients with squamous cell carcinoma (HR, 0.61; 95% CI, 0.42–0.88), although this subgroup finding should be interpreted in the context of the overall trial results [9].

CheckMate 577 therefore established postoperative immune checkpoint blockade as a component of the curative treatment pathway, while leaving unanswered whether immunotherapy would be more effective if administered before surgery, when the primary tumor and regional lymph-node disease remained present [37,38]. Neoadjuvant treatment may provide an opportunity to engage the immune system in the presence of an intact tumor, providing a biological rationale for moving immunotherapy into the preoperative setting [37,38].

### 5.2. Early Development of Neoadjuvant Immunochemotherapy

Early prospective studies have investigated the feasibility and activity of adding ICIs to neoadjuvant treatment for ESCC. The Japanese FRONTiER (JCOG1804E) study evaluated nivolumab in combination with different chemotherapy regimens, including CF, DCF, and FLOT (fluorouracil, leucovorin, oxaliplatin, and docetaxel). Among 37 patients, the reported pCR rates were 7.7% with CF, 33.3% with DCF, and 41.7% with FLOT. The reported 3-year progression-free survival rates were 66.6%, 83.3%, and 91.7%, respectively, with a 3-year OS rate of 82.4% [40,41]. These results are preliminary and should be interpreted cautiously because of the small cohorts and differences in follow-up.

The phase II NICE study evaluated neoadjuvant camrelizumab combined with nab-paclitaxel and carboplatin. Among the 51 patients who underwent surgery, the pCR rate was 39.2%, and the R0 resection rate was 98% [42]. In a subsequent analysis of patients with clinical N2–3 disease from the NICE study, the 2-year OS and recurrence-free survival rates were 78.1% and 67.9%, respectively [43]. Although these findings provide encouraging evidence of activity in patients with more advanced nodal disease, the single-arm design does not establish superiority over conventional neoadjuvant treatment.

NICE-2 is a separate phase II study designed to compare camrelizumab combined with chemotherapy, camrelizumab combined with nCRT, and nCRT alone [44]. The cited publication describes the study protocol; therefore, its results should not be presented as established clinical evidence.

The phase II KEYSTONE-001 study investigated pembrolizumab combined with chemotherapy as neoadjuvant treatment. Among 46 patients included in the efficacy analysis, the major pathological response (MPR) and pCR rates were 72% and 41%, respectively. At a median follow-up of 27.2 months, the reported 2-year OS and DFS rates were 91% and 89% [10]. These results are promising, but the small, non-randomized study design limits comparisons with other regimens and does not establish a survival advantage over standard neoadjuvant treatment [10,45].

### 5.3. From Pathological Response to Survival Evidence

More recent studies have begun to evaluate neoadjuvant immunochemotherapy in randomized trials. In the phase III ESCORT-NEO/NCCES01 trial, camrelizumab combined with chemotherapy was associated with higher pCR rates than chemotherapy alone. The reported pCR rates were 28.0% and 15.4% in the two camrelizumab-containing groups, compared with 4.7% in the chemotherapy-alone group [11]. However, mature survival outcomes were not available in the initial report.

The phase III HCHTOG1909 trial evaluated perioperative toripalimab combined with neoadjuvant paclitaxel and cisplatin, followed by adjuvant toripalimab, compared with neoadjuvant chemotherapy alone. In the first interim analysis of 252 patients, the pCR rate was 18.6% in the toripalimab group and 4.6% in the chemotherapy-alone group. The 1-year event-free survival (EFS) rates were 77.9% and 64.3%, respectively (HR, 0.62; 95% CI, 0.39–1.00; *p* = 0.050), while the 1-year OS rates were 94.1% and 83.0% (HR, 0.48; 95% CI, 0.24–0.97; *p* = 0.037) [46]. A subsequent planned interim report involving 401 patients showed pCR rates of 26.1% with perioperative toripalimab and 6.2% with chemotherapy alone [47]. These findings provide encouraging randomized evidence of improved pathological response with perioperative toripalimab. However, the later interim report primarily updated the pathological response findings and did not establish the durability of a survival benefit. Longer follow-up is required to determine whether the improvement in pathological response translates into sustained clinical benefit.

The randomized phase III SCIENCE trial directly compares three neoadjuvant strategies: chemotherapy plus sintilimab, nCRT plus sintilimab, and nCRT alone. In the reported interim analysis, the pCR rates were 18.0%, 57.3%, and 49.0%, respectively [48]. Although these findings demonstrate differences in pathological response across the treatment groups, the relative long-term efficacy of the three strategies remains undetermined because mature EFS results were not reported in the cited abstract.

Taken together, these studies demonstrate the growing role of immunotherapy-based combinations in neoadjuvant ESCC treatment. Nevertheless, pCR and MPR are measures of pathological response and should not be equated with established long-term clinical benefit. For several contemporary regimens, EFS and OS data remain immature, and the maturity of survival evidence varies across studies. Pathological response rates should therefore be interpreted separately from survival outcomes, and differences in pCR or MPR rates alone should not be used to conclude that one regimen is superior to another. Key clinical trials of immunotherapy in resectable ESCC are summarized in Table 2.

### 5.4. A New Neoadjuvant Treatment Paradigm

The emergence of immunotherapy adds a third dimension to neoadjuvant treatment intensification, alongside intensified systemic chemotherapy and nCRT. However, several questions remain unresolved. The optimal chemotherapy backbone and treatment duration have not been established, and differences in pathological response among immunochemotherapy regimens have not yet been shown to translate into differences in survival [11,49,50].

Recent randomized studies have begun to move the field beyond feasibility and pathological response toward evaluating clinically meaningful outcomes. Nevertheless, the available evidence remains heterogeneous, and the maturity of survival data varies across trials [11,46,47,48]. Differences in pathological response rates should therefore not be interpreted as evidence of superiority between regimens, particularly when studies differ in chemotherapy backbones, treatment duration, use of radiotherapy, and perioperative treatment design.

Several important questions remain unresolved. The optimal chemotherapy backbone and treatment duration have not been established, and the relative roles of nCRT and immunochemotherapy require further clarification. In parallel, predictive biomarkers, including PD-L1 expression, tumor and immune characteristics, and dynamic molecular markers, require further validation in the neoadjuvant setting [10,51,52,53,54].

Beyond tumor-related factors and molecular biomarkers, patient-related characteristics may also influence the efficacy and tolerability of neoadjuvant immunochemotherapy. In a retrospective study of patients with ESCC receiving neoadjuvant immunotherapy plus chemotherapy, changes in skeletal muscle index during treatment were associated with pathological response and postoperative complications [55]. These findings suggest that body composition and skeletal muscle status may be relevant when assessing treatment response and perioperative risk. However, further validation in larger prospective cohorts is needed to determine their clinical utility in treatment selection and perioperative management.

Ultimately, the next stage of development may extend beyond choosing among fixed treatment regimens. Integrating baseline tumor characteristics with serial response assessment and MRD evaluation could support response-adapted strategies. However, treatment escalation, de-escalation, or omission of surgery based on these assessments remains investigational and requires prospective validation. The central challenge is to establish strategies that improve long-term oncological outcomes while avoiding unnecessary treatment burden.

## 6. Future Perspectives: Toward Organ Preservation

The increasing number of neoadjuvant treatment options for resectable ESCC has expanded therapeutic possibilities but has also highlighted the importance of accurately assessing treatment response and residual disease. Rather than uniformly intensifying treatment, an important future goal is to identify patients who achieve a sufficiently durable response after neoadjuvant therapy and may therefore be candidates for organ-preserving strategies [37,38,54,56].

### 6.1. Improving the Assessment of Residual Disease

The development of organ-preserving strategies depends on accurate identification of residual disease after neoadjuvant treatment. Conventional response assessment using CT, endoscopy, and biopsy can be limited by treatment-related fibrosis, inflammation, and edema, and an apparent local complete response does not necessarily exclude residual nodal or systemic disease [57,58].

The preSANO study demonstrated that multimodal assessment, including endoscopic bite-on-bite biopsy, endoscopic ultrasonography, and imaging, can improve detection of residual disease after nCRT [58]. However, clinically occult disease may remain despite a negative clinical assessment. The preSINO study further investigated the potential role of ctDNA in this setting and showed that post-nCRT ctDNA positivity was associated with a higher risk of subsequent distant metastasis [57].

ctDNA may therefore provide complementary molecular information beyond conventional clinical assessment. As a minimally invasive marker of MRD, persistent ctDNA after neoadjuvant treatment may identify residual systemic disease that is not readily detected by imaging, endoscopy, or pathological assessment [56,57,59]. In a prospective cohort of patients with locally advanced ESCC, integration of personalized ctDNA assessment with conventional clinical evaluation improved the detection of non-pCR, while postoperative MRD positivity was associated with poorer survival outcomes [59]. These findings provide a potential molecular basis for incorporating ctDNA into response assessment and future organ-sparing strategies.

Nevertheless, ctDNA/MRD should currently be considered complementary rather than definitive evidence of residual disease status. Its clinical utility for determining subsequent treatment or safely omitting surgery requires prospective validation [56,57,59].

Response assessment after nCT or immunochemotherapy also warrants specific consideration. In ESCC, endoscopic response after nCT has been associated with pathological response and prognosis, and a nationwide validation study confirmed its prognostic significance [60]. However, endoscopic response does not necessarily establish the absence of residual primary or nodal disease. Similarly, although imaging modalities such as 18F-fluorodeoxyglucose positron emission tomography/computed tomography have shown potential for predicting pathological response after neoadjuvant immunochemotherapy, their ability to reliably identify patients in whom esophagectomy can be safely omitted remains unestablished [61]. Thus, response assessment criteria developed after nCRT should not automatically be extrapolated to nCT or immunochemotherapy. Prospective studies are needed to establish treatment-specific multimodal assessment strategies and validate their safety before these approaches are used to guide organ-preserving management.

### 6.2. From Clinical Complete Response to Organ Preservation

Improved assessment of residual disease has raised the possibility of avoiding esophagectomy in carefully selected patients who achieve a clinical complete response (cCR) after neoadjuvant treatment. Active surveillance is therefore being investigated as an alternative to immediate surgery in patients with a favorable clinical response. Although active surveillance is not itself a neoadjuvant treatment strategy, its development is closely linked to the accuracy of post-neoadjuvant response assessment.

Importantly, cCR should not be equated with a pCR. Although endoscopy with biopsies and imaging can provide complementary information about treatment response, residual viable tumor may remain despite apparently negative clinical assessments. This distinction is particularly important when considering active surveillance or other organ-preserving strategies, because an incorrect classification of response could delay potentially curative surgery. Accordingly, response assessment should integrate endoscopic findings and biopsies with appropriate imaging modalities, while molecular biomarkers may provide additional information as their clinical utility is established. Emerging multimodal approaches, including the integration of imaging and digital pathology features, may further improve response prediction; however, these approaches remain investigational and require prospective validation before they can guide decisions to omit surgery [62].

Clinical evidence specifically in ESCC has also begun to emerge. In a retrospective study of 94 patients with ESCC who achieved cCR after chemoradiotherapy, 37 underwent active surveillance and 57 underwent planned surgery. Three-year OS was comparable between the groups (50% vs. 59%), although the non-randomized design and differences in baseline characteristics limit direct comparison [63].

The phase III SANO trial subsequently provided prospective randomized evidence that active surveillance can be evaluated as an alternative management strategy after nCRT. In patients with cCR, 2-year OS was 74% with active surveillance and 71% with standard surgery, meeting the prespecified non-inferiority criterion in the modified intention-to-treat analysis [64]. However, ESCC represented only a minority of the study population, limiting direct extrapolation to ESCC [64]. Thus, SANO supports the feasibility of response-based organ-preserving management but does not establish active surveillance as a universal alternative to esophagectomy for ESCC.

Organ preservation can also be considered in the setting of definitive chemoradiotherapy (dCRT), in which surgery is reserved for selected patients with residual or recurrent disease. In the JCOG0909 study, a single-arm confirmatory trial in patients with clinical stage II/III esophageal cancer, dCRT followed by selective salvage treatment yielded a 3-year overall survival rate of 74.2%. Salvage surgery was performed in 25 patients (27%), with an R0 resection rate of 76%; however, grade 3–4 early postoperative complications occurred in 20% of patients undergoing salvage surgery [65]. These findings support the feasibility of a dCRT-based strategy with selective salvage treatment, while highlighting the potential morbidity of salvage surgery. The ongoing NEEDS trial is designed to compare nCRT followed by planned esophagectomy with dCRT followed by surveillance and salvage esophagectomy when required in patients with operable, locally advanced ESCC. The trial evaluates overall survival and health-related quality of life, but comparative results are not yet available from the cited protocol [66]. This strategy should be distinguished from active surveillance after nCRT, as the initial treatment approach and the role of surgery differ between the two settings.

Importantly, the potential value of organ preservation extends beyond avoidance of surgery itself. In a health-related quality-of-life analysis of the SANO trial, active surveillance was associated with more favorable patient-reported outcomes in several domains during follow-up, supporting the potential functional benefits of avoiding esophagectomy in appropriately selected patients [67].

### 6.3. ctDNA/MRD and Response-Adapted Organ Preservation

The emerging evidence suggests that organ preservation may depend on integrating multiple modalities rather than relying on a single marker. Endoscopy with targeted biopsy, imaging, pathological response, and ctDNA/MRD may provide complementary information on local and systemic residual disease [56,57,58,59]. A recent review similarly emphasized that organ-sparing strategies after neoadjuvant treatment require accurate response assessment and structured surveillance because cCR does not necessarily indicate pCR [68].

In this context, ctDNA/MRD may have particular value because it can provide molecular information that complements anatomical and endoscopic assessment. The prospective ESCC study by Liu et al. showed that combining ctDNA with conventional clinical assessment increased the sensitivity for detecting non-pCR after nCRT and that postoperative MRD status was associated with subsequent outcomes [59]. These findings support further investigation of ctDNA/MRD as a component of response-adapted treatment and organ-sparing strategies, although they do not yet establish ctDNA as a criterion for omitting surgery. Importantly, the prognostic value of ctDNA/MRD should be distinguished from its predictive value for treatment selection. Although ctDNA positivity may indicate residual molecular disease and an increased risk of recurrence, current evidence is insufficient to demonstrate that ctDNA-guided treatment escalation or de-escalation, or omission of surgery, improves clinical outcomes.

The key challenge is therefore not simply whether surgery can be omitted, but whether residual disease can be detected with sufficient accuracy to identify patients in whom omission of surgery is oncologically safe. Future prospective studies should establish standardized criteria for cCR, define the role of ctDNA/MRD alongside conventional assessment, and determine appropriate surveillance and salvage strategies. Pathological response should continue to be evaluated as an intermediate endpoint, while disease-free and OS remain essential measures of clinical benefit [11,46,54,56,57,59].

The future development of neoadjuvant treatment for ESCC may not be defined solely by further treatment intensification. Rather, an important goal is to determine whether accurate clinical, endoscopic, imaging, pathological, and molecular assessment can identify patients who achieve durable disease control without esophagectomy. If this can be achieved, the treatment paradigm may shift from neoadjuvant therapy followed by surgery toward definitive organ-preserving management. Such a shift could create opportunities to reconsider treatment regimens, duration, and dose intensity, as treatment strategies would no longer need to be designed primarily around maintaining fitness for major surgery and minimizing toxicity that could compromise surgical safety. However, these possibilities remain hypothetical and require prospective evaluation. Current evidence for active surveillance is derived primarily from patients achieving a clinical complete response after nCRT, and its applicability to ESCC and to newer neoadjuvant immunochemotherapy regimens requires further investigation.

The evaluation of these strategies should extend beyond oncological outcomes to include long-term functional outcomes and patient-reported quality of life. Swallowing function, nutritional status, and physical functioning are particularly relevant when weighing the potential benefits of intensified multimodal treatment against the functional consequences of esophagectomy and the potential benefits of organ preservation [69]. Prospective studies should therefore assess these outcomes alongside disease-free and OS to determine whether response-adapted organ-preserving strategies can achieve durable cancer control while preserving function and quality of life.

## 7. Conclusions

The evolution of neoadjuvant treatment for resectable ESCC has been driven by efforts to overcome the limitations of surgery alone and conventional perioperative therapy. Treatment has progressed from postoperative chemotherapy to preoperative chemotherapy, intensified systemic therapy, and neoadjuvant chemoradiotherapy, with ICIs introducing new possibilities for curative-intent treatment. Each strategy has addressed persistent challenges in systemic disease control, locoregional control, and treatment-related burden.

Despite these advances, the optimal neoadjuvant strategy for resectable ESCC remains incompletely defined. The relative roles and optimal sequencing of intensified chemotherapy, chemoradiotherapy, and immunochemotherapy require further clarification, as does the identification of patients most likely to benefit from each approach. Future progress will depend not only on treatment efficacy but also on accurate assessment of treatment response and residual disease. Emerging approaches, including ctDNA/MRD assessment, may complement conventional imaging, endoscopy, and biopsy to improve response evaluation and risk stratification, although their clinical utility requires prospective validation.

An important future direction is the development of response-adapted treatment strategies, including organ preservation in carefully selected patients. Active surveillance after a clinical complete response has emerged as a potential alternative to immediate esophagectomy, supported by prospective randomized evidence from the SANO trial; however, its applicability to ESCC and its long-term oncological safety require further evaluation. Ultimately, the goal is to identify patients who can achieve durable disease control without esophagectomy while ensuring timely salvage treatment for residual or recurrent disease. Prospective studies should evaluate not only oncological outcomes but also long-term functional outcomes and quality of life to establish whether response-adapted strategies can maintain cancer control while reducing treatment burden.

## Figures and Tables

**Table 1:** Landmark randomized trials shaping the evolution of perioperative and post-neoadjuvant treatment for resectable esophageal squamous cell carcinoma.

Trial	Population	Treatment	Comparator	Key Outcome	Clinical Contribution
***Neoadjuvant treatment trials***					
JCOG9204 [3]	Localized thoracic ESCC	Surgery → postoperative CF ×2	Surgery alone	5-year DFS, 55% vs. 45%; OS, 61% vs. 52%	Supported postoperative chemotherapy to reduce recurrence.
JCOG9907 [4]	cStage II/III thoracic ESCC	Preoperative CF ×2 → surgery	Surgery → postoperative CF ×2	5-year OS, 55% vs. 43%; HR, 0.73	Established preoperative chemotherapy as the standard in Japan.
CROSS [5]	Resectable esophageal/GEJ cancer; ESCC and AC	Carboplatin + paclitaxel + 41.4 Gy → surgery	Surgery alone	Median OS, 49.4 vs. 24.0 months; R0 resection, 92% vs. 69%	Established nCRT as an effective multimodal strategy, particularly in ESCC.
NEOCRTEC5010 [6]	Locally advanced ESCC	Vinorelbine + cisplatin + 40 Gy → surgery	Surgery alone	5-year OS, 59.9% vs. 49.1%; HR, 0.74	Provided randomized evidence supporting nCRT in ESCC.
JCOG1109 NExT [7]	cStage IB/II/III ESCC	DCF → surgery	CF → surgery; CF + RT → surgery	3-year OS, 72.1% vs. 62.6%; HR, 0.68 (DCF vs. CF)	Supported systemic in-tensification with DCF over conventional CF.
NeoRes [24,25]	Resectable esophageal/GEJ cancer; ESCC and AC	nCRT → surgery	nCT → surgery	Higher pathologi-cal response and R0 resection with nCRT; no signifi-cant OS benefit	Showed greater tumor regression with nCRT without a clear survival advantage.
CMISG1701 [26]	cT3–4aN0–1M0 ESCC	nCRT → minimally invasive esophagectomy	nCT → minimally invasive esophagectomy	pCR, 27.7% vs. 2.9%; 3-year OS, 64.1% vs. 54.9%; HR, 0.82	Questioned the survival benefit of nCRT over nCT despite higher pCR.
ESCORT-NEO/NCCES01 [11]	Resectable locally advanced ESCC	Camrelizumab + chemotherapy → surgery	Chemotherapy → surgery	pCR, 28.0% vs. 4.7%; MPR, 59.1% vs. 20.9%	Provided phase III evidence that adding an ICI to nCT improves pathological response.
***Post-neoadjuvant/adjuvant therapy***					
CheckMate 577 [9]	Resected stage II/III esophageal/GEJ cancer with residual disease after nCRT	Adjuvant nivolumab	Placebo	Median DFS, 22.4 vs. 11.0 months; HR, 0.69	Established adjuvant nivolumab after nCRT and surgery for residual disease.

CF, cisplatin plus fluorouracil; DCF, docetaxel plus cisplatin plus fluorouracil; DFS, disease-free survival; ESCC, esophageal squamous cell carcinoma; GEJ, gastroesophageal junction; AC, adenocarcinoma; HR, hazard ratio; MPR, major pathological response; nCT, neoadjuvant chemotherapy; nCRT, neoadjuvant chemoradiotherapy; OS, overall survival; pCR, pathological complete response; RT, radiotherapy; ICI, immune checkpoint inhibitor.

**Table 2:** Key clinical trials of immunotherapy in resectable esophageal squamous cell carcinoma.

Trial	Phase	Treatment Setting	Immunotherapy Strategy	Pathological Response/Key Findings	Survival Evidence
CheckMate 577 [9]	III	Adjuvant after nCRT and R0 resection with residual disease	Nivolumab vs. placebo	Not applicable (adjuvant trial).	Median DFS, 22.4 vs. 11.0 months; HR, 0.69.
FRONTiER (JCOG1804E) [40,41]	II	Neoadjuvant	Nivolumab + chemotherapy	pCR: 7.7% (CF), 33.3% (DCF), and 41.7% (FLOT).	3-year PFS: 66.6%, 83.3%, and 91.7%, respectively; overall 3-year OS, 82.4%. Preliminary results from small cohorts.
NICE [42,43]	II	Neoadjuvant	Camrelizumab + chemotherapy	pCR, 39.2%; R0 resection, 98% (among 51 patients who underwent surgery).	2-year OS, 78.1%; RFS 67.9% (in the clinical N2–3 subgroup).
NICE-2 [44]	II	Neoadjuvant	Camrelizumab + chemotherapy vs. camrelizumab + nCRT vs. nCRT alone	Clinical results not available in the cited protocol.	Survival benefit not established.
KEYSTONE-001 [10]	II	Neoadjuvant	Pembrolizumab + chemotherapy	MPR, 72%; pCR, 41%;	2-year OS, 91%; DFS, 89%. Randomized confirmation needed.
ESCORT-NEO/NCCES01 [11]	III	Neoadjuvant	Camrelizumab-based chemotherapy regimens vs. chemotherapy alone	pCR: 28.0% and 15.4% in the camrelizumab-containing groups vs. 4.7% in the control group.	Mature survival benefit was not established in the initial report.
HCHTOG1909 [46,47]	III	Perioperative	Toripalimab + neoadjuvant chemotherapy, followed by adjuvant toripalimab, vs. neoadjuvant chemotherapy alone	pCR, 26.1% vs. 6.2% in the later interim report.	Initial interim analysis: 1-year EFS, 77.9% vs. 64.3%; 1-year OS, 94.1% vs. 83.0%. Longer follow-up is needed.
SCIENCE [48]	III	Neoadjuvant	Chemotherapy + sintilimab vs. nCRT + sintilimab vs. nCRT alone	pCR: 18.0%, 57.3%, and 49.0%, respectively.	Mature EFS results were not reported in the cited interim abstract.

nCRT, neoadjuvant chemoradiotherapy; pCR, pathological complete response; CF, cisplatin plus fluorouracil; DCF, docetaxel plus cisplatin plus fluorouracil; FLOT, fluorouracil, leucovorin, oxaliplatin, and docetaxel; MPR, major pathological response; DFS, disease-free survival; HR, hazard ratio; PFS, progression-free survival; OS, overall survival; RFS, recurrence-free survival; EFS, event-free survival. Note: pCR and MPR are not validated surrogates for long-term survival after neoadjuvant immunochemotherapy for ESCC. For HCHTOG1909, the later interim report primarily updated pathological response; survival estimates are from the initial interim analysis.

## Data Availability

No new data were created or analyzed in this study. Data sharing is not applicable to this article.

## Abbreviations

The following abbreviations are used in this manuscript:

ESCC	Esophageal squamous cell carcinoma
nCRT	Neoadjuvant chemoradiotherapy
nCT	Neoadjuvant chemotherapy
ctDNA	Circulating tumor deoxyribonucleic acid
MRD	Molecular residual disease
CF	Cisplatin plus fluorouracil
DFS	Disease-free survival
OS	Overall survival
HR	Hazard ratio
ICIs	Immune checkpoint inhibitors
DCF	Docetaxel, cisplatin, and fluorouracil
CF-RT	Cisplatin plus fluorouracil combined with radiotherapy
pCR	Pathological complete response
PD-1	Programmed cell death 1
PD-L1	Programmed cell death 1-ligand 1
CPS	Combined positive score
FLOT	Fluorouracil, leucovorin, oxaliplatin, and docetaxel
MPR	Major pathological response
EFS	Event-free survival
cCR	Clinical complete response
dCRT	Definitive chemoradiotherapy
